# Impact of disease activity on quality of life and EQ-5D-3L score in myasthenia gravis: results from the Swedish MG registry

**DOI:** 10.1007/s00415-025-13298-4

**Published:** 2025-08-07

**Authors:** Malin Petersson, Jing Wu, Fredrik Berggren, Ingrid Schager, Fredrik Piehl, Susanna Brauner

**Affiliations:** 1 Neuroimmunology Unit, Department of Clinical Neurosciences, Karolinska Institutet, 17176 Stockholm, Sweden; 2 Institute of Environmental Medicine, Karolinska Institutet, Stockholm, Sweden; 3 UCB, Copenhagen, Denmark; 4 UCB, Stockholm, Sweden; 5 Department of Neurology, Karolinska University Hospital, Stockholm, Sweden

**Keywords:** Myasthenia gravis, Quality of life, Disease activity, EQ-5D, Patient reported outcomes

## Abstract

**Introduction:**

Myasthenia gravis (MG) is an autoimmune disease causing motor fatiguability. The impact of MG on quality of life (QoL) is incompletely studied. Here, we explore the impact of disease activity on QoL.

**Methods:**

Using the Swedish nationwide MG registry, 150 MG cases with concomitant EQ-5D-3L and MG–ADL, QMG and/or MG–QoL-15 scores were identified. An EQ-5D score was derived from EQ-5D-3L using the UK time trade-off tariff and compared to a Swedish reference population. Longitudinal analysis assessing the impact of change in MG–ADL on QoL was performed on data from the randomized-controlled RINOMAX trial.

**Results:**

EQ-5D score was negatively correlated with MG–ADL, QMG and MG–QoL-15 (*R* =  − 0.48, − 0.33, and − 0.66, respectively; *p* < 0.01). The usual activities dimension deviated most from reference population, although all dimensions were significantly impacted. Lower EQ-5D score was observed already at low-to-moderate disease activity, MG–ADL 3–5 points, compared to reference (0.71 [SD 0.23] vs. 0.81 [SD 0.04]; *p* = 0.01), and was further decreased with higher disease activity. Longitudinal analyses revealed a significant decrease of EQ-5D score per point increase in MG–ADL (*β* –0.050 [95%CI –0.077, –0.023]; *p* < 0.001). Most impact on EQ-5D score was observed by limb and respiratory items (limb *β* − 0.17 [95%CI − 0.26, − 0.071]; *p* = 0.001, respiratory *β* − 0.21 [95%CI − 0.39, − 0.031]; *p* = 0.022), while bulbar symptoms showed no effect on EQ-5D score.

**Conclusions:**

We observed a considerably decreased EQ-5D score already at low disease activity in MG patients, worsening with increasing disease activity. This indicates an unmet medical need that warrants improved treatment and care.

## Introduction

Myasthenia gravis (MG) is an autoantibody mediated chronic disease characterized by periods of relapse and remission. Patients experience extensive muscular fatigability and the disease can potentially be life-threatening [1]. Mortality rates have decreased substantially in the last century to low levels at present, but an excess mortality is still observed [2–5]. Furthermore, existing evidence suggests a considerable impact of MG on daily life activities [6, 7]. In MG, both generic and disease specific tools have been used to evaluate quality of life. Generic QoL scores may be used to compare a distinct patient population to other groups, whereas disease specific scores might capture features of the disease that generic scores do not [8].

Several studies have indicated an association between disease activity and poorer QoL. For example, a recent multinational study with 841 patients collecting self-reported measures via an app, observed a substantial impact of MG disease activity on generic QoL [9] and an inverse correlation between generic QoL and disease activity was reported in a Danish cohort of 178 patients [10]. Based on a nationwide Swedish survey of 1,077 patients, we observed self-reported symptoms associated with an unsatisfactory disease state in almost half of the cohort [6]. Furthermore, in a recent Danish study, one-third of generalized MG patients reported an unacceptable symptom state (patient’s acceptable symptom score [PASS]), associated with significantly lowered both generic and MG-specific QoL scores [11]. In a smaller study of 56 treatment refractory patients, poorer MG-specific QoL was reported compared to non-refractory patients, further indicating an influence of disease activity on QoL [12]. Finally, poor generic QoL has been linked both to non-MG specific factors such as age, female sex, higher BMI, and lower educational attainment, as well as MG-specific factors such as generalized disease and being prescribed multiple immunosuppressive drugs [6, 13–15].

The EuroQol 5-Dimension (EQ-5D) is a questionnaire assessing quality of life based on the impact on five dimensions of health [16]. It is designed to be generic and is used to compare health-related QoL across different diseases, also representing a simple concise tool for both patients and clinicians. EQ-5D is commonly used to calculate utility, a measure developed to assess the overall health state of a person [17], which may aid the evaluation of the cost-effectiveness of interventions and allow for comparison across diseases.

The objective of this study was to describe EQ-5D scores in Swedish MG patients compared to the general population in Sweden, and to investigate the relationships across different levels of disease activity, both at baseline and longitudinally using the MG–activities of daily life (MG–ADL), quantitative myasthenia gravis measure (QMG), and the disease specific MG–Quality of Life-15 measure (MG-QoL-15).

## Methods

### Patient and data

This retrospective study was based on data from the voluntary, nationwide Swedish MG-registry (MGreg), in which data on diagnostic workup, treatment, and disease activity scores on patients with a physician-verified diagnosis of MG are collected longitudinally, typically at outpatient visits. MGreg also includes the patient-reported scores MG-ADL, EQ-5D-3L, MG-QoL-15 and PASS, but not information on comorbidities. MGreg is publicly funded and was initiated in 2011, although data from before 2011 has been retrospectively entered at some centers. Data extraction for this study was made on the 4th of June 2024, at which point 1,429 patients were actively followed in the registry.

All MG patients aged 18 years or older, who had at least one registration of EQ-5D-3L were identified. Data on concomitant scores of MG–ADL, MG–QoL-15, and QMG was collected, if available. Date of first recorded EQ-5D-3L was defined as baseline. Clinical characteristics were summarized including sex, age at onset and baseline, diagnostic delay, disease duration at baseline**,** disease subtype, antibody status, and pharmacological treatment. MG–ADL score items were used to classify patients’ symptoms as ocular, bulbar, limb, and respiratory.

The RINOMAX trial was a Swedish randomized-controlled (RCT) trial assessing the efficacy of rituximab compared to placebo as add-on to standard of care in new-onset generalized MG over 48 weeks (NCT02950155) [18]. In total 47 patients were included and subject's scores for MG–ADL and EQ-5D-3L were recorded six times during the study period. The data from the RINOMAX trial was included to enable longitudinal analyses with standardized follow-up time.

### Quality-of-life scores

Data on QoL was collected using the EQ-5D-3L questionnaire, where a person’s self-rated QoL is assessed based on the impact of the five dimensions mobility, self-care, usual activities, pain and discomfort, and anxiety and depression [16]. An EQ-5D score is generated based on the response to the dimensions, where a higher EQ-5D score indicates better QoL and a value of 1 indicates perfect health [19]. Two versions of the EQ-5D exist: one with three levels for each item (EQ-5D-3L) and, since 2009, one with five levels (EQ-5D-5L), which was introduced to limit ceiling effects and increase informativity [20]. In the Swedish MG registry, the EQ-5D-3L version was used until 2023, after which the EQ-5D-5L version was implemented.

At present, one Swedish EQ-5D value set, as well as Swedish reference data using the United Kingdom (UK) value set is publicly available [21, 22]. To more readily compare our data to existing national reference values, we used the UK time-trade off (TTO) value set using the ‘EQ. 5D’ R-package to generate an EQ-5D score [23]. Swedish reference data on the frequency of the general population reporting moderate–severe problems by each dimension was used to compare to the study cohort [22].

The 15-item MG–Quality-of-Life (MG-QoL-15) scale consists of 15 questions each with five levels (0–4p). It is a patient reported scale, which assesses the physical, psychological and social aspects of MG ranging from 0 to 60 points, where higher scores indicate worse QoL [8].

PASS is a one question measure enquiring if the patient believes they have an acceptable disease state or not. In MG, the cutoff for an unacceptable disease state has been established at MG–ADL 3 points, MG–QoL-15 score 9 points, and QMG score 8 points, respectively [24].

### Disease activity scores

MG–ADL is an eight-item, four-level patient-reported disease activity measure ranging from 0 to 24 points with higher scores indicating increased disease activity [25]. A score of 0 or 1 has been used to denote minimal disease manifestations [26]. Furthermore, a score of 2 or less has been suggested as the estimated threshold for PASS [24]. There is no well-established cutoff to denote more severe or active disease. However, in recent pivotal RCTs on MG treatment, such as RAISE and REGAIN, a score of 6 points or more was used as an inclusion criterion to capture patients with active disease; similarly, the ADAPT trial included patients with an MG–ADL score of 5 points or more [27–29].

The QMG is a physician-reported score consisting of 13 items with four levels, and total scores ranging from 0 to 39, where higher points indicate higher disease activity [30]. QMG captures neck-weakness and general fatigability more readily than MG–ADL [31].

### Data analysis and statistics

Descriptive data are presented as counts and frequencies for categorical variables or means and standard deviation (SD) for continuous variables. Statistical significance was considered for *p* values < 0.05. For patients with more than one registration of EQ-5D, the first recorded score was used for correlation analyses between the EQ-5D score and QMG, MG–ADL or MG–QoL-15, to avoid decreased validity due to changed internal standards over time. Data preparation and statistical analyses were conducted using R statistical software (version 4.1.3).

EQ-5D scores at different levels of disease activity measured by MG–ADL or QMG, and disease specific QoL measured by MG–QoL-15, were compared to Swedish reference values based on age and sex using a two-tailed *t* test.

To assess the impact of each dimension of the EQ-5D in MG patients we used reference data with summary statistics of moderate–severe problems (*n* and percent), calculating *p* values using chi-square test, and visualizing the results in a grouped bar graph. Correlations of EQ-5D and disease activity, including subdomains of MG–ADL, were assessed using Spearman rank correlation due to the monotonic rather than strictly linear pattern of the data. To visualize EQ-5D score across different levels of MG-specific scores (MG–ADL, QMG and MG–QoL), we used local regression (LOESS) on baseline scores. To further dissect the impact of subdomains of MG–ADL on EQ-5D score, we used all available MG–ADL scores during follow-up.

To determine the association between disease activity and the EQ-5D score either at baseline or the longitudinal change, we used data from patients included in the RINOMAX trial, which offered a more structured and frequent follow-up compared to the real-world data of MGreg. For total MG–ADL and its subdomains (ocular, bulbar, limb and respiratory) the change in ADL score (ΔADL) was calculated by subtracting the baseline score from the follow-up. Linear mixed-effect models were used to estimate *β*-coefficients of baseline ADL and average ΔADL with 95%CI with adjustment of potential confounders age at baseline, sex, disease duration, and treatment. The between-subject and within-subject effects of time-varying change in ADL were estimated by including the mean of ΔADL and standard deviation of ΔADL for each participant, respectively. Time was calculated for the weeks from the baseline assessment. We considered a random intercept and slopes with an unstructured variance matrix. The linear mixed-effects models were conducted in StataMP (18, StataCorp LLC, College Station, TX).

Due to the exploratory nature of the study, no correction for multiple testing was performed.

## Results

### Baseline characteristics

At data extraction, 538 EQ-5D-3L scores registered between December 2013 and March 2023 were identified. In total, 150 unique patients had at least one EQ-5D-3L score with other scores reported simultaneously, and were, therefore, included in the study. Of these, 47 patients were from the RINOMAX study (Table 1) [18]. Patients were predominantly male (55%) with a mean age of 63 (SD 15) years and a mean disease duration of 9 (SD 10) years, and 63% were classified as late-onset MG. Mean EQ-5D score in the whole cohort was 0.70 (SD 0.27; range − 0.484–1), vs. 0.81 (SD 0.04) in the Swedish reference population. Approximately one-third (*n* = 55, 37%; 78% overlap with the RINOMAX cohort) of subjects had baseline within 1 year after onset and were defined as new onset.

**Table 1:** Baseline patient characteristics

Characteristic	Overall *N* = 150	Not RINOMAX *N* = 103	RINOMAX *N* = 47	New onset *N* = 55	EOMG, AChR + *N* = 29	LOMG, AChR + N = 68
Sex, female	67 (45%)	53 (51%)	14 (30%)	18 (33%)	22 (76%)	12 (18%)
Age at onset, yrs	55 (19)	50 (18)	64 (17)	63 (16)	30 (12)	68 (8)
Age at baseline, yrs	63 (15)	62 (14)	64 (17)	64 (16)	45 (14)	71 (9)
Disease duration, yrs	9 (10)	12 (11)	2 (1)	0.35 (0.5)	16 (14)	4 (4)
Diagnostic delay, yrs	1 (3)	1 (4)	0 (1)	0 (0)	2 (5)	1(1)
Anti-AChR positive	109 (89%)	64 (84%)	45 (98%)	49 (98%)	29 (100%)	68 (100%)
Anti-MuSK positive	3 (13%)	3 (18%)	0 (0%)	0 (0%)	1 (3%)	0 (0%)
Seronegative	11 (9%)	10 (13%)	1 (2%)	1 (2%)	-	-
*Subgroup*						
EOMG	41 (30%)	34 (39%)	7 (15%)	7 (13%)	29 (100%)	-
LOMG	85 (63%)	46 (52%)	39 (83%)	45 (83%)	-	68 (100%)
TAMG	9 (7%)	8 (9%)	1 (2%)	2 (4%)	-	
*Treatment*						
AChEi	108 (72%)	66 (64%)	42 (89%)	47 (85%)	24 (83%)	55 (81%)
Corticosteroids	38 (25%)	20 (19%)	18 (38%)	22 (40%)	5 (17%)	23 (34%)
IST	74 (49%)	55 (53%)	19 (40%)	22 (40%)	14 (48%)	34 (50%)
Biologics	41 (31%)	41 (46%)	0 (0%)	5 (10%)	8 (30%)	16 (25%)
IST + Biologics	93 (70%)	74 (82%)	19 (45%)	26 (53%)	16 (59%)	43 (68%)
Longitudinal scores	90 (60%)	43 (42%)	47 (100%)	47 (85%)	18 (62%)	50 (74%)
Number of EQ-5D-3L	4 (3)	2 (1)	8 (2)	6 (3)	3 (3)	5 (3)
EQ-5D score	0.70 (0.27)	0.73 (0.24)	0.64 (0.32)	0.65 (0.31)	0.78 (0.16)	0.69 (0.31)
Reference EQ-5D score	0.81 (0.04)	0.81 (0.03)	0.82 (0.04)	0.81 (0.04)	0.85 (0.03)	0.80 (0.03)
MG–ADL, points	5 (4)	4 (3)	6 (4)	5 (4)	4 (4)	5 (4)
QMG, points	8 (5)	4 (4)	10 (4)	10 (4)	7 (6)	9 (4)
MG–QoL-15, points	18 (13)	15 (12)	24 (13)	23 (13)	18 (12)	20 (14)

Continuous data presented as mean (SD), and categorical data as *n* (%). AChR, Acetylecholine Receptor. AChEi; Acetylcholine Esterase Inhibitors. Anti-AChR; Anti-Acetylcholine Receptor antibodies. Anti-MuSK; Anti-Muscle Specific Kinase antibodies. EOMG; Early onset MG. LOMG; Late-onset MG. IST; Immunosuppressive therapy (Azathioprine, Ciclosporine, Mycophenolatemofetil). Biologics (Rituximab or Tocilizumab). MG–ADL; myasthenia gravis–activities of daily life. MG–QoL-15; myasthenia gravis–quality of life-15. TAMG; Thymoma-associated MG. QMG; quantitative myasthenia gravis

### Impact of MG in EQ-5D dimensions

**Fig. 1:**
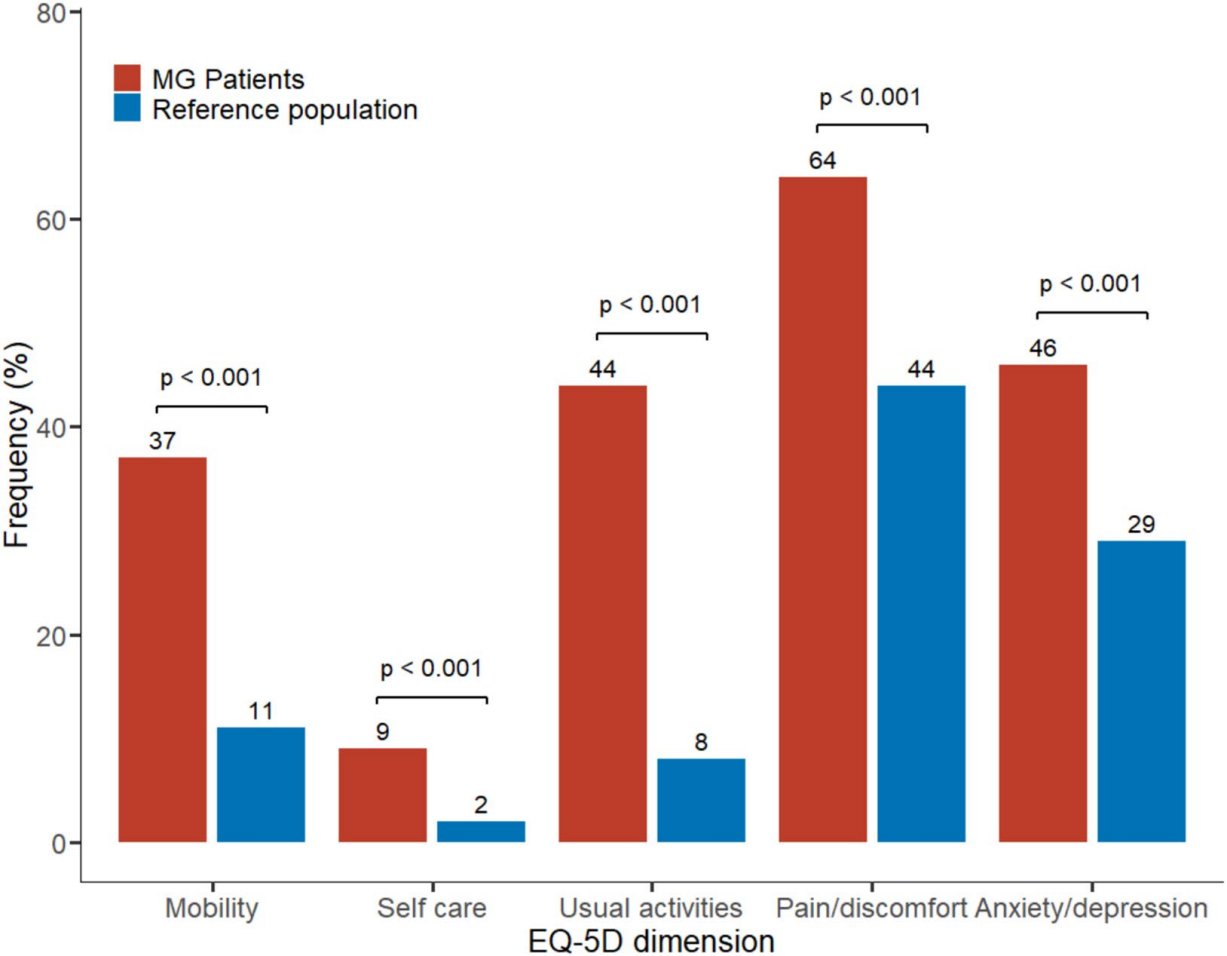
Impact in EQ-5D dimensions in MG patients compared to general population. Proportion of MG patients (*n* = 150) reporting moderate or severe impact in each EQ-5D dimension compared to previously published values from a representative Swedish population (*n* = 3069, Burström et al. 2001). *P* values for comparison from chi-squared test

We first sought to compare the frequency of moderate–severe problems in the five different EQ-5D dimensions between MG patients and a Swedish reference population. All five dimensions were significantly more impacted in MG patients compared to reference population (*p* < 0.001, Fig. 1**)**. The largest difference was observed in the usual activities dimension (44% compared to 8%, respectively; *p* < 0.001), followed by self-care (9.3% compared to 1.9%, respectively; *p* < 0.001).

### Correlation between disease activity and QoL measures

**Fig. 2:**
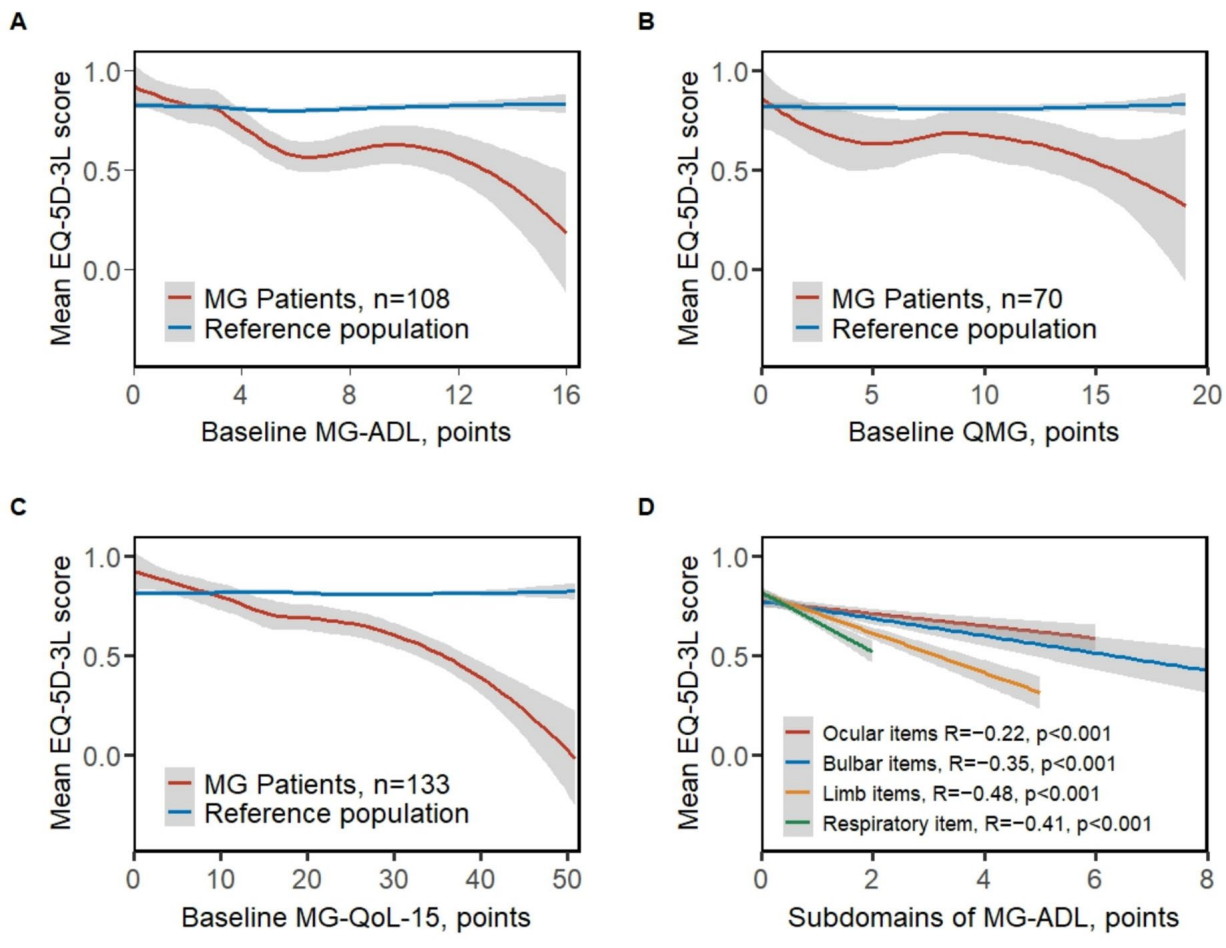
EQ-5D score correlated with MG specific scores. EQ-5D-3L-derived EQ-5D score correlated with **A** baseline MG–ADL *R* =  − 0.48, *p* < 0.001, *n* = 108, **B** baseline QMG *R* =  − 0.33, *p* =.003, *n* = 70, **C** baseline MG–QoL-15 *R* =  − 0.66, *p* < 0.001, *n* = 133, **D** subdomain scores for MG–ADL (*n* = 108) ocular, limb, bulbar and respiratory items. Correlation coefficients obtained by Spearman method. EQ-5D score from reference with the same sex and age distribution as the patients is shown as a reference. Local regression (LOESS) is used to summarize the underlying data points in panels A–C and linear regression to visualize data points in panel D. MG–ADL; myasthenia gravis–activities of daily life. MG–QoL-15; myasthenia gravis–quality of life-15. QMG; quantitative myasthenia gravis

We thereafter investigated the correlation of EQ-5D with other scores and observed a stronger association with the patient-reported MG–ADL (*R* − 0.48; *p* < 0.001) and MG–QoL-15 scores (*R* − 0.66; *p* < 0.001), than with the physician-evaluated QMG (*R* − 0.33; *p* = 0.003) (Fig. 2A–C). Interestingly, when correlating MG–ADL or MG–QoL-15 with the EQ-5D score (Fig. 2A, C), the intercept of the MG patients and reference populations occurred at approximately 2 and 8 points of the MG–ADL and MG–QoL-15 scales, respectively, which corresponds well with the respective PASS cutoffs previously proposed by Mendoza et al. [24].

### Comparison of groups with different disease activity

To further dissect the relationship between the EQ-5D score and disease activity or MG–QoL-15, patients were stratified into groups based on previously defined cutoffs for PASS (MG–ADL ≤ 2 points, QMG ≤ 7 points, and MG–QoL-15 ≤ 8 points; Table 2) [24]. Patients with scores above the cutoff reported significantly worse EQ-5D scores than those below (MG–ADL; 0.86 [SD 0.16] vs. 0.65 [SD 0.28], *p* < 0.001 and MG–QoL-15;0.87 [SD 0.17] vs. 0.62 [SD 0.28; *p* < 0.001). The QMG cutoff was less precise, but an average difference of 0.10 of the EQ-5D score was observed between the groups (0.73 [SD 0.27] vs. 0.61 [SD 0.32]; *p* = 0.1). Further dissecting the impact of disease activity on the EQ-5D score, we observed a significant decrease at mild–moderate disease activity, MG–ADL 3–5 points vs. ≤ 2 points, (*p* < 0.003). We also investigated the effect of even higher disease activity by identifying patients with MG–ADL 6 − 9 points and ≥ 10 points, respectively, where similar EQ-5D scores were observed (0.60 [SD 0.28] and 0.58 [SD 0.39], respectively).

**Table 2:** EQ-5D score stratified on disease measures

	*N*	Patient EQ-5D score	Reference EQ-5D score	*p* value (comparison to reference population)	*p* value (comparison between PASS positive and negative groups based on Mendoza et al.)
*MG–ADL*					
0	14	0.94 (0.11)	0.83 (0.03)	0.001	
≤ 2	33	0.86 (0.16)	0.82 (0.04)	0.17	< 0.001
≥ 3	75	0.65 (0.28)	0.81 (0.03)	< 0.001	
3 − 5	35	0.71 (0.23)	0.81 (0.04)	0.01	
6 − 9	27	0.60 (0.28)	0.81 (0.03)	< 0.001	
≥ 10	13	0.58 (0.39)	0.82 (0.02)	0.03	
*QMG*					
≤ 7	32	0.73 (0.27)	0.82 (0.04)	0.07	0.1
≥ 8	38	0.61 (0.32)	0.82 (0.04)	< 0.001	
*MG–QoL-15*					
≤ 8	42	0.87 (0.17)	0.81 (0.04)	0.03	< 0.001
≥ 9	91	0.62 (0.28)	0.81 (0.03)	< 0.001	

Baseline MG–ADL, QMG, MG–QoL-15 and EQ-5D scores (*n* = 108, *n* = 70 and *n* = 133, respectively) stratified based on acceptable symptom states (MG–ADL ≤ 2 points; QMG ≤ 7 points; MG–QoL-15 ≤ 8 points) and commonly used MG–ADL cutoffs for moderate and high disease activity. Expected EQ-5D score values for a sex and age-matched UK reference population based on the UK time trade-off tariff. EQ-5D score presented as mean (SD). MG–ADL; myasthenia gravis–activities of daily life. MG–QoL-15; myasthenia gravis–quality of life-15. QMG; quantitative myasthenia gravis

### Impact of different muscle groups on EQ-5D score at baseline and longitudinally

To explore the impact of different muscle on EQ-5D score, we correlated MG–ADL subdomain scores for ocular, bulbar, respiratory, and limb items with the EQ-5D score using all available scores (Fig. 2D). Interestingly, respiratory and limb items displayed stronger correlations to EQ-5D (*R* − 0.41 and − 0.48, respectively; both *p* < 0.001) than ocular and bulbar items (*R* − 0.22 and *R* − 0.35, respectively; both *p* ≤ 0.001), indicating that a one-point change in limb and respiratory muscle groups would impact the EQ-5D score most.

Next, using data from the RINOMAX clinical trial, we explored ΔADL in relation to the longitudinal change in EQ-5D and MG–QoL-15 (Table 3). We observed that a one-point higher ΔADL score was associated with a 0.025-point lowering of the EQ-5D score (*β* − 0.025 [95%CI − 0.051, − 0.001]; *p* = 0.057) and with a 2.0-point higher MG–QoL-15 score (*β* 2.0 [95%CI 0.86, 3.07]; *p* = 0.001) at baseline. During the follow-up, a one-point increase in ΔADL was associated with a 0.050-point faster rate of reduction in the EQ-5D score (*β* − 0.050 [95%CI − 0.077, − 0.023]; *p* < 0.001) and a 3.5-point faster rate of increase in MG–QoL-15 (*β* 3.5 [95%CI 2.30, 4.60]; *p* < 0.001). Stratifying by muscle group, a significant impact from limb items was observed on EQ-5D score (*β* –0.095 [95%CI –0.16, –0.026]; *p* = 0.007) at baseline. MG–QoL-15 was significantly associated with Δocular (*β* 3.66 [95%CI 1.20, 6.13]; *p* = 0.004), Δrespiratory (*β* 9.06 [95%CI 1.64, 16.48]; *p* = 0.017) and Δlimb (*β* 5.59 [95%CI 2.52, 8.66]; *p* < 0.001) items of ADL at baseline. Furthermore, we observed impact on the EQ-5D score primarily by Δrespiratory (*β* – 0.21 [95%CI –0.39, –0.031]; *p* = 0.022) and Δlimb symptoms (*β* –0.17 [95%CI –0.26, –0.071]; *p* = 0.001), but also Δocular (*β* –0.078 [95%CI –0.15, –0.010]; *p* = 0.024). Similarly, longitudinal change of MG–QoL-15 was mainly affected by Δrespiratory (*β* 16.35 [95%CI 7.27, 25.44]; *p* < 0.001) and Δlimb symptoms (*β* 11.10 [95%CI 6.88, 15.32]; *p* < 0.001), and to a lesser extent Δocular (*β* 5.44 [95%CI 2.18, 8.69]; *p* = 0.001) and Δbulbar symptoms (*β* 3.9 [95%CI 0.51, 7.24]; *p* = 0.024) (Table 3).

**Table 3:** Trajectory of EQ-5D score and MG–QoL-15 over 48-week follow-up by disease activity using mixed-effect models

	EQ-5D score		MG–QoL-15	
	*β* (95%CI)	*p* value	*β* (95%CI)	***p*** value
*MG–ADL total score*				
ADL at baseline	–0.025 (–0.051, 0.001)	0.057	1.96 (0.86, 3.07)	0.001
Average ΔADL	–0.050 (–0.077, –0.023)	0.000	3.45 (2.30, 4.60)	0.000
*MG–ADL subdomain scores in separate models*				
Ocular at baseline	–0.035 (–0.086, 0.017)	0.19	3.66 (1.20, 6.13)	0.004
Average ΔADL-ocular	–0.078 (–0.15, –0.010)	0.024	5.44 (2.18, 8.69)	0.001
Bulbar at baseline	0.003 (–0.077, 0.083)	0.94	0.11 (–3.74, 3.96)	0.96
Average ΔADL-bulbar	–0.050 (–0.12, 0.019)	0.16	3.88 (0.51, 7.24)	0.024
Respiratory at baseline	–0.14 (–0.29, 0.009)	0.065	9.06 (1.64, 16.48)	0.017
Average ΔADL-respiratory	–0.21 (–0.39, –0.031)	0.022	16.35 (7.27, 25.44)	0.000
Limb at baseline	–0.095 (–0.16, –0.026)	0.007	5.59 (2.52, 8.66)	0.000
Average ΔADL-limb	–0.17 (–0.26, –0.071)	0.001	11.10 (6.88, 15.32)	0.000

Models were adjusted for age, sex, disease duration, and treatment. Each participant was assessed with EQ-5D-3L six times over the 48-week period. ΔADL = ADL score during follow-up – baseline ADL score. MG–ADL; myasthenia gravis–activities of daily life. MG–QoL-15; myasthenia gravis–quality of life-15

## Discussion

In this study, we derived an EQ-5D score from EQ-5D-3L, assessing the health-related quality of life in 150 MG patients from the nationwide Swedish MG-registry, and made correlations with MG–ADL, QMG and MG–QoL-15. Compared to Swedish reference data, MG patients reported considerably more problems across all EQ-5D dimensions. Furthermore, for MG-specific scales, we observed an inverse correlation, with higher disease specific scores associated with lower EQ-5D score. Interestingly, even patients with low disease activity experienced significantly decreased EQ-5D score, which is concerning and underscores the need for more active treatment interventions in a larger proportion of MG patients. Finally, we modeled change of disease activity on EQ-5D score over time and observed a significant impact of MG–ADL, specifically associated with respiratory and limb symptoms, highlighting symptoms in need of special attention in clinical practice.

The impact of MG across all EQ-5D dimensions was significant compared to controls, particularly in usual activities, followed by self-care and mobility. Similarly, in the MyRealWorld MG study, usual activities was the dimension with the highest frequency of moderate–extreme problems reported [9]. We further observed a significant drop in EQ-5D score in patients already at low disease activity (MG–ADL > 2 points), as also observed in the MyRealWorld MG study [9]. Interestingly, the mean EQ-5D score of the MG patients included in the present study was lower than EQ-5D scores previously reported in other diseases, such as colorectal cancer, brain tumors, malignant melanoma, and diabetes type I and II [32]. This is somewhat surprising, as MG is typically not a progressive disease and with only slightly increased mortality rates [4]. We speculate that the fluctuating nature of MG causes unpredictability which affects daily life and social interactions that may contribute to the reduced QoL of people with MG [33]. This is in agreement with the results from the MyRealWorld MG study, in which patients had noticeable burden of disease despite being on effective treatment [34]. However, in MyRealWorld MG data were self-reported by patients via a smartphone app, introducing a risk of a biased population enriched for participants with high disease activity. While this limitation may also apply to the Swedish MGreg, it is a health care run registry covering approximately half of all prevalent MG patients in Sweden, thereby providing generalizability. However, when comparing to a recent Danish study, the overall EQ-5D score in this study was lower than that found by Kahr Andersen et al., which could be explained by differences in disease activity as they report mean MG–ADL 3 points, compared to 5 points here [11]. Regarding QoL measurements, it is also important to consider the occurrence of a ‘response shift’ in which patients change their internal standards for QoL as a consequence of having lived with, and thereby coped with, a disease for longer [35, 36]. In this study, even though we used the earliest available EQ-5D-3L score, the average disease duration was 9 years at baseline, when any response shift should already have taken place, thereby mainly reflecting a chronic state of MG.

In previous studies, the cutoffs for both the identification of minimal disease activity and acceptable symptom state (PASS) have been estimated to MG–ADL ≤ 2 points [24, 26]. Interestingly, however, we observed significantly higher EQ-5D scores in patients with MG–ADL 0 points than in the reference population. In the group with MG–ADL ≤ 2 points EQ-5D scores similar to the reference population were noted; however, patients with higher disease activity had significantly lower scores. This further emphasizes the clinical relevance of MG–ADL ≤ 2 points as indicative of good disease control. We further observed a tendency toward a plateau-like shape of the EQ-5D score curve at moderate disease activity scores. This has not been described previously, but might reflect the nature of the data set comprising patients with varying disease duration, who may have either decreasing, increasing or stable disease activity that might influence their quality of life. Hence, a one point change of MG–ADL might not bear the same weight at different levels of disease activity and where patients with high scores in single items could have a greater impact on the EQ-5D score, compared to patients with low disease activity in multiple muscle groups [9]. Unfortunately we could not address this hypothesis further, due to a limited number of patients with more severe scores, which calls for future larger prospective studies from disease onset.

In the longitudinal analysis we observed that a change in respiratory and limb items impacted both the EQ-5D score and MG–QoL-15 more than ocular and bulbar items. If replicated, this raises the question whether more attention to these symptoms, by, for example, weighting of the items of MG–ADL, is warranted to make the assessment more informative in relation to QoL. It is also of interest that we did not observe any association of bulbar symptoms to the EQ-5D score, neither at baseline nor longitudinally, while for MG–QoL-15, a comparatively small increase was noted in the longitudinal analysis and no association was noted at baseline. While our results must be interpreted with caution due to the low number of patients and the exploratory nature of the study and the risk of selection bias, we believe that this could be due to the predominantly mild symptoms exhibited by included patients. Similar results implying limb symptoms rather than ocular and bulbar affecting QoL have been reported previously using MG impairment index domains, strengthening the reliability of our findings [37]. However, results should be interpreted with caution as this could reflect the inability of EQ-5D to capture the impact of such symptoms.

A limitation of the study is that we used EQ-5D-3L, which has lower ceiling effects than EQ-5D-5L, and provides somewhat less informativity especially in individuals with multi-morbidity [38, 39]. As the EQ-5D-5L has now been implemented in the Swedish MGreg, this provides a possibility to extend this study in the future. Furthermore, we were unable to compare our data to a Swedish reference material using a Swedish value set, but we still could compare it to a Swedish population, where scores were generated using the UK tariff. Data on comorbidities and psychological health status were unfortunately not available in MGreg, which is a major limitation to the study. We were further unable to identify generalized and ocular MG patients with high precision for stratified analyses, as MGFA scores are not collected. Despite being a comparatively large MG patient cohort, a lack of concomitant scores resulted in a loss of power and granularity of the analyses and a bias toward patients included in the RINOMAX trial (*n* = 46, 32% of the cohort). Furthermore, we only analyzed longitudinal data from the RINOMAX trial, as the time between scores was too heterogenous in the non-RINOMAX patients.

In conclusion, in this study we find a significant impact on the EQ-5D score compared to the general population, already at low–moderate disease activity. Furthermore, there was a significantly larger impact of respiratory and limb symptoms on QoL measures compared to bulbar and ocular symptoms. Our findings indicate an unmet medical need at low-to-moderate disease activity, in turn raising the question if also patients with milder disease activity scores should be included in clinical trials to develop more effective treatment algorithms.

## Declarations

### Data availability

Anonymized data generated for the study are available from the corresponding author on reasonable request.
